# Facilitators and Barriers to Implementing Interventions to Prevent Musculoskeletal Disorders in Blue-Collar Workers: A Scoping Review

**DOI:** 10.1007/s10926-023-10162-y

**Published:** 2024-01-13

**Authors:** Suzan Mooren-van der Meer, Denise J. M. Smit, Nathan Hutting, Wim van Lankveld, Josephine Engels, Michiel Reneman, Thomas Pelgrim, J. Bart Staal

**Affiliations:** 1 Musculoskeletal Rehabilitation Research Group, School for Allied Health, HAN University of Applied Sciences, Nijmegen, The Netherlands; 2 Centre for Nutrition, Prevention and Health Services, National Institute for Public Health and the Environment, Bilthoven, The Netherlands; 3 Research Group Occupation and Health, School of Organisation and Development, HAN University of Applied Sciences, Nijmegen, The Netherlands; 4 Department of Rehabilitation Medicine, University Medical Center Groningen, University of Groningen, Groningen, The Netherlands; 5 Research Department Emergency and Critical Care, Faculty of Health and Social Studies, HAN University of Applied Sciences, Nijmegen, The Netherlands; 6 Radboud Institute for Health Sciences, IQ Healthcare, Radboud University Medical Centre, Nijmegen, The Netherlands

**Keywords:** Health inequalities, Musculoskeletal health, Scoping review, Work, Prevention, Consolidated framework for implementation research

## Abstract

**Purpose:**

Blue-collar workers generally have less healthy lifestyles, poorer health, and a lower life expectancy than white-collar workers. At least in part this may be attributed to their work and working conditions. Employers increasingly provide interventions to improve health and wellbeing and prevent musculoskeletal disorders. However, they often do not reach blue-collar workers. The aim of this scoping review was to identify the facilitators for and barriers to implementing such interventions among blue-collar workers.

**Methods:**

A scoping review in which the study population of the selected studies consists of blue-collar workers (≥ 18 years old) in paid employment. Furthermore, included studies should report facilitators and barriers to implementing interventions to prevent musculoskeletal disorders. The literature search was conducted in six databases. The resulting studies were extracted with the help of the updated Consolidated Framework for Implementation Research.

**Results:**

15 articles were included; these were reviews, intervention studies, qualitative studies and process evaluations. A main facilitator was a participatory approach, which involves the blue-collar worker in the entire process of defining, developing, and implementing a multidimensional preventive intervention. The main barriers on the worker level were unfavorable worker characteristics and unsupportive behavior/attitudes. The main barriers on the organization level were a culture with a high production standard, a hierarchical culture, inflexible work, and an unsupportive attitude from the employer.

**Conclusion:**

This review showed the multifaceted nature of implementation. A tailored implementation plan that involves the stakeholders (including workers) is important.

## Introduction

About 15–50% of the world’s adult population suffers from musculoskeletal disorders (MSDs) [1–4]. The wide range in prevalence can be partly explained by the extensive range of definitions for MSDs in scientific literature [4]. The number of people with MSDs is predicted to grow exponentially in the next two decades because of the age distribution of populations and their longevity [5, 6]. MSDs such as back, neck and shoulder pain have a major impact on daily functioning and participation, including work [7]. As a result, MSDs strain the healthcare, employment, and social security systems [5, 6]. MSDs are affected by physical, psychological, and social factors [6, 8].

In the working population, job dissatisfaction, high job demands, and repetitive movements are strongly associated with the occurrence and prognosis of MSDs [3]. This is particularly the case for blue-collar workers: those who predominantly perform manual labor [3, 8, 9]. The prevalence of MSDs among them can be attributed to their work, at least in part [10–12]. On average, they have less healthy lifestyles, poorer health, and a lower life expectancy than white-collar workers [13]. There is a growing awareness of the need to prevent the development of MSDs in the workplace, especially for blue-collar workers [14–17]. There is also a social and ethical urgency to improve health equality and prevent MSDs, particularly in the blue-collar workforce, as they impact the individual, occupational, and social levels of workers [18, 19].

Increasingly, employers are providing interventions (e.g., task rotations, customized training, and knowledge-related interventions) with the aim of improving health and wellbeing and preventing MSDs [14, 16, 19]. However, these interventions often do not reach blue-collar workers and, even if they participate, they drop out earlier and do not comply as well as white-collar workers [20, 21]. Multiple systematic reviews have concluded that interventions for blue-collar workers are not as effective as interventions for white-collar workers [7, 13, 14], but the reasons have not been investigated systematically. Researchers mention reasons related to occupational, individual and cultural factors (e.g., financial constraints, or interventions that are not attractive enough for blue-collar workers) [20, 21]. It is unclear which facilitators and barriers influence the implementation of interventions to prevent MSDs in blue-collar workers, and implementation science can help us investigate this systematically [22].

To this end, it is extremely important to better understand the facilitators and barriers to implementing interventions to prevent MSDs among blue-collar workers. Therefore, this scoping review aims to identify these facilitators and barriers. We will summarize and discuss the results from studies that address facilitators and barriers to implementing preventive interventions, with the hope that they can be used to improve the implementation of interventions to prevent MSDs among blue-collar workers.

## Methods

### Design

A scoping review was used to identify and analyze gaps in knowledge bases. A scoping review has a broader scope than a systematic review [23]: the scoping process is iterative (not linear) and it requires researchers to engage with each stage in a reflective way and, where necessary, to repeat steps to ensure that the literature is covered comprehensively [24]. The review was reported according to the Prisma checklist for scoping reviews [25].

### Information Source and Search

Multiple systematic searches were performed with the help of a medical librarian until June 22, 2022, in the following databases: Medline(Ebsco), Embase.com, PsycInfo(Ebsco), Cinahl plus with full text(Ebsco), Cochrane Central, and Web of Science(Core Collection). The following terms were used (including synonyms and closely related words) as index terms or free-text words to represent the following concepts: “musculoskeletal complaints” AND “blue-collar workers” AND “prevention” AND publication type. No additional filters were used. The full searches are available in “Appendix 1”. All results per database were exported into a single file. The files then were merged and de-duplicated in EndNote using the Bramer method [26]. The reference lists of all included reports and articles were searched for additional studies (snowball method).

### Study Selection

To be included, studies should investigate facilitators and barriers to implementing interventions for primary and secondary prevention of MSD. Furthermore, the study population of the selected studies must consist of blue-collar workers (≥ 18 years old) in paid employment. Published articles from medical, vocational, and social contexts were included. Only more economically developed countries with a human development index of 0.80–1.0 (very high) were included because they have comparable systems comparable to the Netherlands. Included study types were reviews, intervention studies (e.g., randomized controlled trials and cohort studies) and qualitative studies regarding interventions to prevent MSD. To capture the latest evidence, only studies published since 2007 were included. For more detailed information about the eligibility criteria, see “Appendix 2”.

Two authors (SM and DS) independently screened all titles and abstracts acquired from the systematic search using RAYYAN [27]. The authors checked their degree of agreement four times (after 20, 200, 1000, and all studies) and discussed discrepancies until they reached consensus. When consensus was not reached, a third author (JBS) was involved to make a decision. Full texts of all studies were screened by the same two authors to make a final decision about inclusion.

### Data Extraction and Analyses

Results were extracted as follows: author, year of publication, source of origin, study design, sample size, methodology, and variety of intervention. The facilitators and barriers to implementing interventions to prevent musculoskeletal complaints among blue-collar workers were extracted and summarized following the updated Consolidated Framework for Implementation Research (CFIR) [22]. The CFIR guide consists of five domains related to the implementation of interventions: intervention, implementation process, individuals, inner setting, and outer setting (see Table 1 for more information). The framework guidance was used to select the most suitable domain [22]. The first author screened the articles for reported facilitators and barriers. The second author (DS) had research experience with the CFIR guide and helped to select the most suitable domain [28, 29].

**Table 1:** Domains of the consolidated framework for implementation research

Domain	Meaning
Intervention	The intervention that is being implemented: e.g., job rotation, ergonomic tool
Implementation process	The activities and strategies used to implement the intervention
Individuals	The roles and characteristics of individuals: e.g., employees, high-level leaders, opinion leaders, implementation team members and intervention recipients (i.e., workers)
Inner setting	The setting in which the intervention is implemented: e.g., company or industry. There may be multiple inner settings and/or multiple levels within the inner setting
Outer setting	The setting in which the inner setting exists: e.g., city, country or society. There may be multiple outer settings and/or multiple levels within the outer setting

## Results

**Fig. 1:**
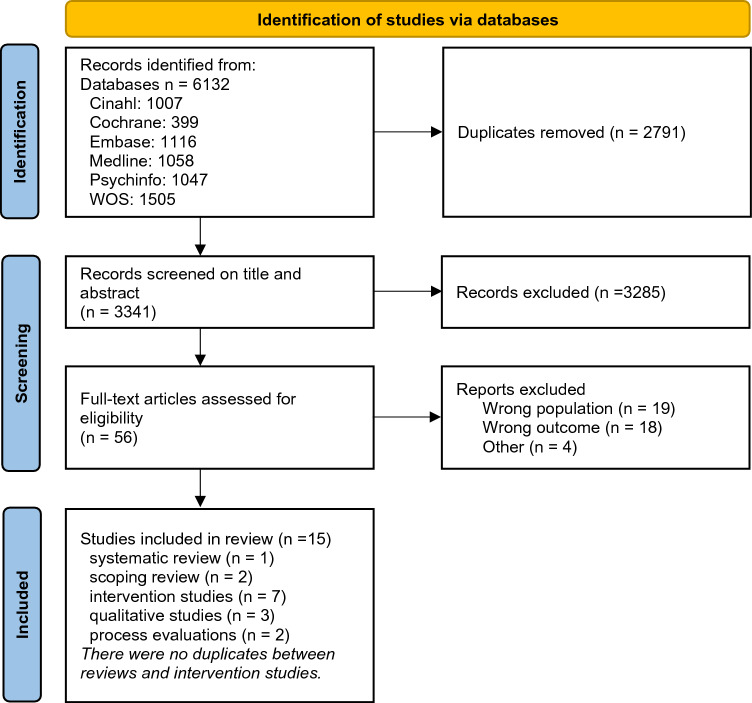
Flow diagram

The search resulted in 3341 abstracts to screen (Fig. 1) on title and abstract. The authors reached consensus for 96% (3224) of the screened titles and abstracts. 117 articles were discussed and, after discussion, six articles were presented to a third author to reach a conclusion. In total, 15 articles were included in this scoping review with full consensus between the two authors after discussion. No additional articles were found by screening the reference lists.

The included articles and their characteristics are summarized in Table 2. They included one systematic review [30], two scoping reviews [12, 31], seven intervention studies (three follow-up studies, two participatory interventions and two pilot studies), [32–38], three qualitative studies [39–41], and two qualitative process evaluations based alongside intervention studies [42, 43]. The main facilitators and barriers of each domain are described in Table 3 and explained in the text if necessary.

**Table 2:** Included studies

References	Study design	Country	Participants	Sample size	Intervention
Bosch et al. (2018) [39]	Qualitative study	Netherlands	Farmworkers	9 workers	Preventive interventions for upper extremity complaints, duration not described
Boyette and Bell (2021) [31]	Scoping review	various	Industrial workers	14 studies; 2681 workers in total	Various interventions (strengthening, stretching, health coaching, work simulation), duration between 4 and 52 weeks
Dale et al. (2016) [37]	Intervention study (participatory)	USA	Construction workers	95 workers	Interactive training for improving ergonomics and knowledge, duration not described
Gyi et al. (2013) [38]	Intervention study (participatory)	UK	Companies with business drivers	4 large companies with > 500 workers each	Co-development of ergonomic activities, duration not described
Jones and James (2018) [34]	Intervention study (pilot)	Australia	Coal miners	27 workers	Task rotation schedule, duration 6 months
Kozak et al. (2019) [12]	Scoping review	various	Hairdressers	44 studies; 4251 workers	Various (exercise, ergonomics, knowledge), duration not described
Kramer et al. (2010) [40]	Qualitative study	Canada	Construction workers	20 companies; workers not described	Various interventions (e.g., ergonomics, tools), duration not described
Leider et al. (2015) [41]	Qualitative study	Netherlands	Construction workers	23 workers	Job rotation, duration not described
May et al. (2008) [33]	Intervention study (single arm, follow-up study)	USA and Australia	Farmworkers	42 workers	Community-based developed ergonomic tool, duration not described
van der Molen et al. (2009) [32]	Intervention study (single arm, follow-up study)	Netherlands	Construction workers	469 workers	Knowledge and ergonomic measures, duration 4.5 years
Oude Hengel et al. (2011) [42]	Process evaluation (randomized controlled trial)	Netherlands	Construction workers	6 companies, 121 workers	Physiotherapy and rest-break tool, duration 6 months
Ouellet and Vézina (2014) [36]	Qualitative study	Canada	Meat processing workers	350 workers	Customized training and education, duration not described
Sell et al. (2015) [35]	Intervention study (single arm, follow-up study)	Denmark	Industrial workers	249 workers	Ergonomic learning program, duration 2.5 years
Padula et al. (2017) [30]	Systematic review	various	Manufacturing industry	14 studies; 889 workers	Job rotation, duration not described
Visser et al. (2018) [43]	Process evaluation (double-arm trial)	Netherlands	Construction workers	12 companies, 277 workers	Face-to-face or e-guidance strategy for a participatory ergonomic intervention, duration 6 months

**Table 3:** Summarizing main facilitators and barriers of each domain related to the Consolidated Framework of Implementation Research

Domain	Facilitators	Barriers
Intervention	Participatory approach [33, 38] Training by employees [12, 30, 36] Multidimensional program with choices for workers [31, 32, 39]	More than three job rotations in a shift [34]
Implementation process	Sufficient knowledge about intervention and goals [39] Powerful implementation strategies [32]	Indirect way of recruiting workers [43] Lack of stability in workers who participate [37] Lack of employer support [39]
Individual	Workers’ autonomy, facilitative work behavior and attitude [12, 41]	Unfavorable work characteristics and unsupportive behavior / attitude [39, 41]
Inner setting	Positive organizational climate with engagement from higher management [12, 35, 37, 41, 42]	Organizational culture with a high production standard, hierarchical culture and inflexible work [39–41] Employer’s unwillingness to change if there is no visible work disability [39]
Outer setting	–	Recession [42] Environmental conditions [37]

### Intervention-Related Facilitators and Barriers

Intervention-related facilitators and barriers were described in one systematic review [30], one scoping review [31], five intervention studies [32–34, 36, 38], and one qualitative study [39].

The use of a participatory design might be a facilitator because it raises awareness among workers and management and improves communication [33, 38]. With a participatory approach, the design and methodology are flexible and can be adapted to risks such as changes in key personnel, internal politics, organizational structures, and global economics [38].

A second facilitator is using the expertise of experienced employees to develop training content and train workers, so they make the intervention their own [12, 30, 36]. A third possible facilitator is having the intervention consist of multidimensional programs with choices for workers, so the program can be tailored to the risk profile of the individual or the workplace [31, 32, 39].

However, having more than three job rotations in a shift is a barrier to implementation. When using job rotation, it is important that the intervention be feasible and practical (e.g., a maximum of three job rotations during a nine-hour shift) [34].

### Implementation-Process-Related Facilitators and Barriers

Facilitators and barriers related to the implementation process were described in two intervention studies [32, 37], one qualitative study [39] and one process evaluation [43].

Having sufficient knowledge about the intervention and goals can help workers during an intervention [39]. Another facilitator is the use of powerful implementation strategies, in which different implementation strategies were combined in a multifaceted way. For instance, the combination of training (educational strategy) and stimulating collaboration (facilitating strategy) to reduce physical work demands and reduce MSD.) [32].

### Individual-Related Facilitators and Barriers

Individual-related facilitators and barriers were described in one scoping review [12], one intervention study [41], and one qualitative study [39]. Workers’ autonomy in their work and during the intervention, facilitative work behavior and a supportive attitude are facilitators to starting and implementing an intervention [12, 41]. Barriers include unfavorable worker characteristics (e.g., a lack of knowledge about physical work exposures and skills), impeding work behavior (communication and cooperation with employer and colleagues) and an unsupportive attitude from the employer [39, 41].

### Inner-Setting-Related Facilitators and Barriers

Facilitators and barriers related to the inner setting were described in one scoping review [12], two intervention studies [35, 37], three qualitative studies [39–41], and one process evaluation [42].

Overall, organizational culture is an important factor. This includes a supportive organizational climate (e.g., the awareness of employers of adverse physical work demands and a favorable attitude throughout the organization towards prevention of it), job autonomy, favorable job characteristics (e.g., automatic rotation between tasks and/or activities), flexible work processes and the willingness of the employer to change work demands by moving employees from one department to another when starting and implementing an intervention [12, 35, 41]. Additionally, strong, organized, and attentive leadership may facilitate the process and structure of work to allow workers’ voices and suggestions to be incorporated into planning for the work [37]. Working in a smaller company (< 100 employees) and having greater management engagement in the intervention positively influenced the implementation of an intervention on construction worksites [42].

However, an organizational culture with a high production standard, hierarchical culture, or inflexible work process can be a barrier to implementing preventive programs [39, 41]. Additionally, workers who work alone and do not allow themselves to take breaks, and the absence of the employer at the workplace are barriers to starting and implementing an intervention [39]. If the employer makes incorrect assumptions about job changes and shows little willingness to change when there is no visible work disability, preventive interventions are less likely to be implemented [39, 40].

### Outer-Setting-Facilitators and Barriers

Barriers were described in one intervention study [37] and one process evaluation [42]. We found no facilitators for this domain.

The first barrier is that an economic recession can lead to worker dismissal and/or forcing workers to work part-time. This might affect the amount of the intervention workers receive [42]. Another barrier, specific to construction firms, is environmental conditions (e.g., a muddy working environment) and interactions about the intervention with other involved companies that delay the intervention [37].

## Discussion

### Statement of Principal Findings

This scoping review found multiple facilitators and barriers that might be important for implementing interventions to prevent musculoskeletal complaints among blue-collar workers. These facilitators and barriers are related to all five domains of the CFIR. This demonstrates the multifaceted nature of implementation.

In summary, a main facilitator is a participatory approach that involves the worker in the entire process of defining, developing, and implementing a multidimensional preventive intervention. Other main facilitators are powerful implementation strategies.

The main barriers on the workers’ level involve unfavorable worker characteristics (e.g., lack of knowledge about physical work exposures and skills) and unsupportive behavior/attitudes (e.g., impeding communication and cooperation with employer and colleagues). The main barriers on the organizational level are a culture with a high production standard, a hierarchical culture, inflexible work, and an unsupportive attitude.

### Strengths and Weaknesses of the Study

One strength of this study is the use of the recently updated CFIR, a highly cited framework in implementation science that is focused on predicting or explaining facilitators and barriers to implementation effectiveness [22]. Moreover, the search string was systematic and the search was thorough (as shown in “Appendix 1”). The search string led us to find studies with different designs.

A weakness of this study is that the variety of study designs prevented us from assessing the risk of bias in individual studies. Also, despite the systematic search, this scoping review may have overlooked some studies that could have been found by using different key terms or a broader research question.

### Comparison with Scientific Literature

In line with our findings, an evaluation study [44] showed that the results of (implementing) an intervention reflect the intertwined aspects of the intervention, the research, and the local context. Therefore, a continuous dialog between stakeholders is important and draws attention to the social dynamics and shifting circumstances when implementing an intervention [44]. These shifting circumstances (e.g., recession, environmental conditions) can influence the outcome measures, which could be seen as bias [45, 46] or as an authentic process.

In this review, a participatory approach was identified as a facilitator for implementing interventions. Research that enables active involvement by participants is applied in public health and health promotion and is a valuable option for active participation by blue-collar workers and other important stakeholders [44, 47]. The collaborative development, and especially implementation of the intervention, can also bridge the educational knowledge gap between researchers/developers and workers [45]. However, there have been few structured high-quality studies about a participatory approach [46, 48].

This review also identified workers’ attitudes, behavior, and knowledge and skills as important individual factors that influence implementation. In line with our findings, other qualitative studies also identified a negative attitude as an important barrier to implementation [47, 49]. Since workers’ attitudes can act as both a barrier and a facilitator, it is important to ensure that workers have a positive attitude. Involving blue-collar workers in the development and implementation of the intervention can positively contribute to their attitude and the effectiveness of the intervention [50].

Research also showed that job autonomy is essential for worker engagement and beneficial behavior. This might also lead workers to behave in ways that support preventive interventions [51]. Thus, to improve implementation, companies should also focus on and prioritize their workers’ autonomy.

In line with other studies, this scoping review shows the importance of cultural success factors (e.g., knowing and meeting employees’ needs, leadership involvement and continuity of communication) [28, 52]. Other studies identified another barrier: a masculine culture (e.g., discouraging talking about personal topics, such as lifestyle and health) among blue-color workers can have a negative effect on their health behavior, and thus requires a culture change on the organizational level [21].

### Meaning of the Study: Possible Mechanisms and Implications for Clinicians and Policymakers

This review showed that the implementation of an intervention must be tailored to the inner setting of the company and their workers, and a participatory approach can help to create this. This also indicates that the required measurement of outcomes must be tailored to both employers and employees [53]. For example, health outcomes are not always clear for employers and employees. It is important that the assumptions, emphasis and values that health outcomes contain are understandable for both employers and employees [53]. A company that wants to start implementing an intervention needs to analyze the existing facilitators and barriers a priori, create a customized action plan to strengthen some facilitators, and understand the development of the barriers and how to overcome them. This is also important for improving the health of blue-collar workers and reducing health-related inequalities [54].

### Unanswered Questions and Future Research

There is a need for more high-quality studies focused on identifying the facilitators and barriers that influence blue-collar workers’ decisions about participating in health-related interventions and determining how to tailor (the implementation of) interventions to increase their effectiveness. Specifically, how can facilitators be embedded and how can barriers be overcome? There also is a need for study designs that monitor facilitators and barriers, process evaluations, and realistic synthesis (with a focus on understanding the mechanisms by which an intervention works or not) to gain a better understanding of facilitators and barriers to implementation [55].

### Conclusion and Recommendations

In conclusion, this review showed that multiple facilitators and barriers are related to the implementation of interventions to prevent musculoskeletal disorders among blue-collar workers. The CFIR can help to make the multifaceted nature of implementation visible. When a company wants to start implementing an intervention, it is important to first analyze the existing facilitators and barriers a priori, create a customized action plan to strengthen some facilitators, and understand the development of the barriers and how to overcome them.

## Appendix 1

### Medline

S1: MH "Physical Fitness" OR MH "Posture + " OR MH "Exercise" OR MH "Exercise Test + " OR MH "Musculoskeletal Pain" OR MH "Back Pain + " OR MH "Motor Activity" OR TI ((Physical N1 (Fitness OR activit*)) OR posture OR (motor N1 activit*) OR exercis* OR Musculoskeletal OR back OR knee* OR spin* OR Backache* OR (Vertebrogenic N1 Pain N1 Syndrome*) OR Lumbago OR shoulder*) OR AB ((Physical N1 (Fitness OR activit*)) OR posture OR (motor N1 activit*) OR exercis* OR Musculoskeletal OR back OR knee* OR spin* OR Backache* OR (Vertebrogenic N1 Pain N1 Syndrome*) OR Lumbago OR shoulder*) OR SU ((Physical N1 (Fitness OR activit*)) OR posture OR (motor N1 activit*) OR exercis* OR Musculoskeletal OR back OR knee* OR spin* OR Backache* OR (Vertebrogenic N1 Pain N1 Syndrome*) OR Lumbago OR shoulder*)
S2: MH “Service Personnel” OR MH “Technical Service Personnel” OR MH “Domestic Service Personnel” OR MH “Agricultural workers” OR TI ((Blue N1 Collar*) OR housekeeper* OR cashier* OR beekeeper* OR butcher* OR carpenter OR farmworker* OR miner OR miners OR smelter OR police OR (fire N1 fighter) OR military OR soldier* OR (chain N1 saw N1 operator*) OR (child N1 care N1 work*) OR (trades N1 work*) OR painter* OR hairdresser* OR (waste N1 picker) OR sailor* OR (metal N1 worker*) OR moulder* OR welder* OR blacksmith* OR toolmaker* OR (machinery W1 (mechanic* OR repairer*)) OR (handicraft N1 worker*) OR (manual N1 (laborer* OR labourer*)) OR landscaper* OR (Machine W1 operator*) OR Fisherman OR fishermen OR ((truck* OR cab* OR taxi* OR lorry OR delivery OR car OR crane) N1 (driver* OR chauffeur*)) OR ((correctional OR agricultural OR abattoir OR bakery OR Construction OR security OR cleaning OR coal OR production OR factory OR forest OR gardener* OR sex OR shipyard OR (food N1 service) OR industrial OR manufactor* OR migrant* OR railway OR transport* OR warehouse OR hotel OR hospitality OR catering OR mining OR sanitation OR textile OR (distribution N1 (center* OR centre*)) OR ((low OR minimum) N1 (wage* OR income OR salary OR salaries))) N1 (worker* OR staff OR employee* OR personnel OR occupation* OR labor* OR labour*)) OR (low N1 skill* N1 (work* OR occupation* OR labor* OR labour*))) OR AB ((Blue N1 Collar*) OR housekeeper* OR cashier* OR beekeeper* OR butcher* OR carpenter OR farmworker* OR miner OR miners OR smelter OR police OR (fire N1 fighter) OR military OR soldier* OR (chain N1 saw N1 operator*) OR (child N1 care N1 work*) OR (trades N1 work*) OR painter* OR hairdresser* OR (waste N1 picker) OR sailor* OR (metal N1 worker*) OR moulder* OR welder* OR blacksmith* OR toolmaker* OR (machinery W1 (mechanic* OR repairer*)) OR (handicraft N1 worker*) OR (manual N1 (laborer* OR labourer*)) OR landscaper* OR (Machine W1 operator*) OR Fisherman OR fishermen OR ((truck* OR cab* OR taxi* OR lorry OR delivery OR car OR crane) N1 (driver* OR chauffeur*)) OR ((correctional OR agricultural OR abattoir OR bakery OR Construction OR security OR cleaning OR coal OR production OR factory OR forest OR gardener* OR sex OR shipyard OR (food N1 service) OR industrial OR manufactor* OR migrant* OR railway OR transport* OR warehouse OR hotel OR hospitality OR catering OR mining OR sanitation OR textile OR (distribution N1 (center* OR centre*)) OR ((low OR minimum) N1 (wage* OR income OR salary OR salaries))) N1 (worker* OR staff OR employee* OR personnel OR occupation* OR labor* OR labour*)) OR (low N1 skill* N1 (work* OR occupation* OR labor* OR labour*))) OR SU ((Blue N1 Collar*) OR housekeeper* OR cashier* OR beekeeper* OR butcher* OR carpenter OR farmworker* OR miner OR miners OR smelter OR police OR (fire N1 fighter) OR military OR soldier* OR (chain N1 saw N1 operator*) OR (child N1 care N1 work*) OR (trades N1 work*) OR painter* OR hairdresser* OR (waste N1 picker) OR sailor* OR (metal N1 worker*) OR moulder* OR welder* OR blacksmith* OR toolmaker* OR (machinery W1 (mechanic* OR repairer*)) OR (handicraft N1 worker*) OR (manual N1 (laborer* OR labourer*)) OR landscaper* OR (Machine W1 operator*) OR Fisherman OR fishermen OR ((truck* OR cab* OR taxi* OR lorry OR delivery OR car OR crane) N1 (driver* OR chauffeur*)) OR ((correctional OR agricultural OR abattoir OR bakery OR Construction OR security OR cleaning OR coal OR production OR factory OR forest OR gardener* OR sex OR shipyard OR (food N1 service) OR industrial OR manufactor* OR migrant* OR railway OR transport* OR warehouse OR hotel OR hospitality OR catering OR mining OR sanitation OR textile OR (distribution N1 (center* OR centre*)) OR ((low OR minimum) N1 (wage* OR income OR salary OR salaries))) N1 (worker* OR staff OR employee* OR personnel OR occupation* OR labor* OR labour*)) OR (low N1 skill* N1 (work* OR occupation* OR labor* OR labour*)))
S3: MH "Health Education" OR MH "Health Promotion + " OR TI (prevent* OR (health* N1 (promotion OR check OR education*)) OR WHPP OR WOPAP OR (total N1 worker* N1 health*) OR (employee W1 wellness W1 program*) OR ((work* OR staff OR employee* OR personnel OR occupation* OR labor* OR labour*) W1 (health* OR (well N1 being) OR wellness OR (physical N1 activit*) OR exercise*) W1 (program* OR intervention* OR training OR education OR promotion OR activit*)) OR ((worksite OR workplace) N1 (fitness OR sports))) OR AB (prevent* OR (health* N1 (promotion OR check OR education*)) OR WHPP OR WOPAP OR (total N1 worker* N1 health*) OR (employee W1 wellness W1 program*) OR ((work* OR staff OR employee* OR personnel OR occupation* OR labor* OR labour*) W1 (health* OR (well N1 being) OR wellness OR (physical N1 activit*) OR exercise*) W1 (program* OR intervention* OR training OR education OR promotion OR activit*)) OR ((worksite OR workplace) N1 (fitness OR sports))) OR SU (prevent* OR (health* N1 (promotion OR check OR education*)) OR WHPP OR WOPAP OR (total N1 worker* N1 health*) OR (employee W1 wellness W1 program*) OR ((work* OR staff OR employee* OR personnel OR occupation* OR labor* OR labour*) W1 (health* OR (well N1 being) OR wellness OR (physical N1 activit*) OR exercise*) W1 (program* OR intervention* OR training OR education OR promotion OR activit*)) OR ((worksite OR workplace) N1 (fitness OR sports)))
S4: S1 AND S2 AND S3
S5: (MH "Clinical Trials as Topic + " OR ZT "clinical trial" OR ZT "randomized controlled trial" OR MH "Random Allocation" OR (TX ((clini* N1 trial*) OR (singl* N1 blind*) OR (singl* N1 mask*) OR (doubl* N1 blind*) OR (doubl* N1 mask*) OR (tripl* N1 blind*) OR (tripl* N1 mask*) OR (random* N1 allocat*) OR placebo* OR ((waitlist* OR (wait* and list*)) and (control* OR group)) OR "treatment as usual" OR tau OR (control* N3 (trial* OR study OR studies OR group*)))) OR TI randmom* OR AB random* OR SU random*) not (MH "Animals + " NOT MH "Humans + ") NOT (ZT "comment" or ZT "editorial" or ZT "letter"). https://blocks.bmi-online.nl/catalog/401
S6: MH ("Qualitative Research + " OR MH "Focus Groups" OR ZT "interview" OR MH "Interviews as Topic" OR MH "Narration + " OR MH "Personal Narratives as Topic" OR MH "Grounded Theory" OR MH "Observational Studies as Topic" OR ZT "observational study" OR MH "Tape Recording + " OR TI (“thematic analys*” OR “content analys*” OR “focus group*” OR ethnograph* OR ethnograf* OR etnograf* OR “field stud*” OR phenomenolog* OR narration* OR narrative OR “qualitative stud*” OR “qualitative analys*” OR “qualitative research*” OR “qualitative method*” OR multimethodolog* OR “mixed method*” OR observation* OR “grounded theory” OR “audio recording*” OR “tape recording*” OR audiotape* OR ((“semi-structured” OR semistructured OR unstructured OR informal OR “in-depth” OR indepth OR “face-to-face” OR structured OR guide*) AND (interview* OR discussion* OR questionnaire*))) OR AB (“thematic analys*” OR “content analys*” OR “focus group*” OR ethnograph* OR ethnograf* OR etnograf* OR “field stud*” OR phenomenolog* OR narration* OR narrative OR “qualitative stud*” OR “qualitative analys*” OR “qualitative research*” OR “qualitative method*” OR multimethodolog* OR “mixed method*” OR observation* OR “grounded theory” OR “audio recording*” OR “tape recording*” OR audiotape* OR ((“semi-structured” OR semistructured OR unstructured OR informal OR “in-depth” OR indepth OR “face-to-face” OR structured OR guide*) AND (interview* OR discussion* OR questionnaire*))) OR SU (“thematic analys*” OR “content analys*” OR “focus group*” OR ethnograph* OR ethnograf* OR etnograf* OR “field stud*” OR phenomenolog* OR narration* OR narrative OR “qualitative stud*” OR “qualitative analys*” OR “qualitative research*” OR “qualitative method*” OR multimethodolog* OR “mixed method*” OR observation* OR “grounded theory” OR “audio recording*” OR “tape recording*” OR audiotape* OR ((“semi-structured” OR semistructured OR unstructured OR informal OR “in-depth” OR indepth OR “face-to-face” OR structured OR guide*) AND (interview* OR discussion* OR questionnaire*))). https://blocks.bmi-online.nl/catalog/293
S7: (MH " Meta-Analysis as Topic + " or TI (metaanaly* or (meta N1 analy*) or metanaly*) OR AB (metaanaly* or (meta N1 analy*) or metanaly*) OR SU (metaanaly* or (meta N1 analy*) or metanaly*) or MH " MH "Systematic Reviews as Topic" OR MH "Cochrane Library" or TI (prisma or prospero) OR AB (prisma or prospero) OR SU (prisma or prospero) OR TI ((systemati* or scoping or umbrella or structured literature) N3 (review* or overview*)) OR AB ((systemati* or scoping or umbrella or structured literature) N3 (review* or overview*)) OR SU ((systemati* or scoping or umbrella or structured literature) N3 (review* or overview*)) or TI (systematic* N1 review*) OR AB (systematic* N1 review*) OR SU (systematic* N1 review*) or TI ((systemati* or literature or database* or data base*) N10 search*) OR AB ((systemati* or literature or database* or data base*) N10 search*) OR SU ((systemati* or literature or database* or data base*) N10 search*) or TI ((structured or comprehensive* or systematic*) N3 search) OR AB ((structured or comprehensive* or systematic*) N3 search) OR SU ((structured or comprehensive* or systematic*) N3 search) or TI ((literature N3 review) and AB (search* or database* or (data N1 base*))) or TI (((data N1 extraction) or "data source*") and study selection) OR AB (((data N1 extraction) or "data source*") and study selection) or TI ("search strategy" and "selection criteria") OR AB ("search strategy" and "selection criteria") OR SU ("search strategy" and "selection criteria") or TI ("data source" and "data synthesis") OR AB ("data source" and "data synthesis") OR SU ("data source" and "data synthesis") or AB medline or AB pubmed or AB embase or AB cochrane or TI ((critical or rapid) N2 (review* or overview* or synthes*)) or AB (((critical* or rapid*) N3 (review* or overview* or synthes*)) and (search* or database* or data base*)) OR SU (((critical* or rapid*) N3 (review* or overview* or synthes*)) and (search* or database* or data base*)) or TI metasynthes* or AB metasynthes* OR SU metasynthes* or TI "meta synthes*" OR AB "meta synthes*" OR SU "meta synthes*") not (MH "Animals + " NOT MH "Humans + ") NOT (ZT "comment" or ZT "editorial" or ZT "letter")
S8: (TI (nonrandom* or "non-random*" or "quasi-experimental" or crossover or "cross over" or "parallel group*" or "factorial trial") OR AB (nonrandom* or "non-random*" or "quasi-experimental" or crossover or "cross over" or "parallel group*" or "factorial trial") OR SU (nonrandom* or "non-random*" or "quasi-experimental" or crossover or "cross over" or "parallel group*" or "factorial trial") OR ((MH "Prospective Studies + " or MH " MH "Multicenter Studies as Topic" or TI (cohort* or "follow up" or followup or longitudinal* or prospective* or retrospective* or observational* or multicent* or 'multi-cent*' or consecutive*) OR AB (cohort* or "follow up" or followup or longitudinal* or prospective* or retrospective* or observational* or multicent* or 'multi-cent*' or consecutive*) OR SU (cohort* or "follow up" or followup or longitudinal* or prospective* or retrospective* or observational* or multicent* or 'multi-cent*' or consecutive*)) AND (TI (group or groups or subgroup* or versus or vs or compar*) OR AB (group or groups or subgroup* or versus or vs or compar*) OR SU (group or groups or subgroup* or versus or vs or compar*) or AB ("odds ratio*" or "relative odds" or "risk ratio*" or "relative risk*" or aor or arr or rrr) or (AB ("OR" or "RR") N6 CI)) or TI (versus or vs or compar*) or MH "Comparative Studies" or TI (compar* N1 study) OR AB (compar* N1 study) or SU (compar* N1 study) OR MH "Historically Controlled Study")) NOT (MH "Animals + " NOT MH "Humans + ") NOT (ZT "comment" or ZT "editorial" or ZT "letter")
S9: TI ((participat* N1 (approach OR research)) OR (action N1 research)) OR AB (((participat* N1 (approach OR research)) OR (action N1 research)) OR SU ((participat* N1 (approach OR research)) OR (action N1 research))
S10: S4 AND S5
S11: S4 AND S6
S12: S4 AND S7
S13: S4 AND S8
S14: S4 AND S9

S15: S10 OR S11 OR S12 OR S13 OR S14

Total: 922

## Appendix 2: Screening Eligibility Criteria

**Table:**

Inclusion criteria (according to PCC question: population, concept, context)	Exclusion criteria
Population: Blue-collar workers (≥ 18 years old) in paid employment. A blue-collar worker is a working class person who performs manual labor (which may be skilled or unskilled)	White-collar workers, higher educated workers (university educated)
Concept: The facilitators and barriers of any intervention to prevent primary and secondary musculoskeletal complaints Barriers and facilitators: Factors are considered to be facilitators if their presence promotes workers’ participation in an intervention to promote healthier behavior Factors are considered to be barriers if they impede workers’ participation in an intervention to promote healthier behavior Primary and secondary prevention: To prevent musculoskeletal pain before it occurs (primary) or reduce its impact (secondary) Musculoskeletal complaints: Back, neck and shoulder pain or complaints Intervention: An action of process of intervening in health behavior Outcome: related to barriers and facilitators. Quantitative aspects related to musculoskeletal health (workplace health promotion / health education / prevention / physical fitness / posture / exercise / motor activity)	Tertiary prevention (the prevention of complications in people who have already developed disease and in whom disease prevention is no longer an option) Implementation of interventions that merely focus on safety (avoiding accidents) Strength, body mass index, work absenteeism, safety
Context: published articles from medical, vocational and social contexts Only the more economically developed countries with a human development index of 0.80 to 1.0 (very high) (United States, Canada, Japan, Australia, New Zealand and all the countries of Europe)	Interventions outside the blue-collar work setting, not related to musculoskeletal complaints
Study type: Reviews, intervention studies, randomized controlled trials or cohort studies, qualitative studies of interventions	Cross-sectional studies, case studies
Type of publication: Scientific peer-reviewed, published and listed in PubMed, Embase, Psych info, Cinahl, or Web of Science	
Publication date: 2007–2022	
Language: English	
